# Apolipoprotein B48, the Structural Component of Chylomicrons, Is Sufficient to Antagonize *Staphylococcus aureus *Quorum-Sensing

**DOI:** 10.1371/journal.pone.0125027

**Published:** 2015-05-05

**Authors:** Bradley O. Elmore, Kathleen D. Triplett, Pamela R. Hall

**Affiliations:** Department of Pharmaceutical Sciences, University of New Mexico College of Pharmacy, Albuquerque, New Mexico, United States of America; University of Liverpool, UNITED KINGDOM

## Abstract

Serum lipoproteins (LP) are increasingly being recognized as dual purpose molecules that contribute to both cholesterol homeostasis and host innate defense. In fact, very low LP levels are associated with increased risk of bacterial infection in critically ill patients. In this respect, we reported that apolipoprotein B100 (apoB100), the 4536 amino acid structural protein of very low density lipoprotein (VLDL) produced by the liver, limits *Staphylococcus aureus* pathogenesis. *S*. *aureus* uses quorum-sensing (QS) via the accessory gene regulator (*agr*) operon and an autoinducing peptide (AIP) to coordinate expression of over 200 virulence genes. ApoB100 prevents *agr* activation by binding and sequestering secreted AIP. Importantly, human serum LP are produced not only by the liver, but are also produced by enterocytes, in the form of chylomicrons, during uptake of dietary lipids. In contrast to apoB100 in VLDL, human enterocytes use apoB48, the N-terminal 2152 amino acids (48%) of apoB100, as the structural component of chylomicrons. Interestingly, enteral feeding of critically ill patients has been associated with decreased risk of infectious complications, suggesting chylomicrons could contribute to host innate defense in critically ill patients when serum LP production by the liver is limited during the acute phase response. Therefore, we hypothesized that apoB48 would be sufficient to antagonize *S*. *aureus* QS. As expected, isolated apoB48-LP bound immobilized AIP and antagonized *agr*-signaling. ApoB48- and apoB100-LP inhibited *agr* activation with IC50s of 3.5 and 2.3 nM, respectively, demonstrating a conserved AIP binding site. Importantly, apoB48-LP antagonized QS, limited morbidity and promoted bacterial clearance in a mouse model of *S*. *aureus* infection. This work demonstrates that both naturally occurring forms of apolipoprotein B can antagonize *S*. *aureus* QS, and may suggest a previously unrecognized role for chylomicrons and enterocytes in host innate defense against *S*. *aureus* QS-mediated pathogenesis.

## Introduction

Serum lipoproteins have historically had two primary functions: 1) to transport cholesterol and other insoluble lipids from their source to peripheral tissues to be used for cell membrane assembly, steroid production and fuel (termed forward cholesterol transport), and 2) to transport excess cholesterol from the tissues to the liver for clearance (reviewed in [1]). In recent years, however, serum lipoproteins have become increasingly recognized for their contribution to host innate defense [2–4]. Extremely low serum lipoprotein levels (hypolipoproteinemia) are associated with increased risk of bacterial infection in critically ill patients such as those experiencing trauma [4–7]. Reduced serum lipoprotein levels in these patients is in part due to the acute phase response (APR) and decreased lipoprotein released from the liver (reviewed in [4, 6]). However, in addition to very low-density lipoprotein (VLDL) and high density lipoprotein (HDL) produced by the liver, human serum lipoproteins include chylomicrons produced by intestinal enterocytes during the uptake of dietary lipids. Therefore, the absence of chylomicron production by the gut in patients unable to receive oral feeding may also contribute to hypolipoproteinemia and an increased risk of infection. Although results are varied, some studies have shown that enteral feeding of critically ill patients, which preserves the contribution of the gut to nutritional processing, is associated with reduced risk of infectious complications compared to parenteral (intravenous) feeding [8, 9]. This suggests that, beyond their role in lipid transport, chylomicrons produced by enterocytes may also contribute to limiting the pathogenesis of bacterial infection. Although VLDL, LDL (produced by lipase reduction of VLDL), HDL and their components are being increasingly recognized as host innate effectors, much less is known about the contribution of chylomicrons and their components to protection against bacterial pathogenesis.

**Fig 1:**
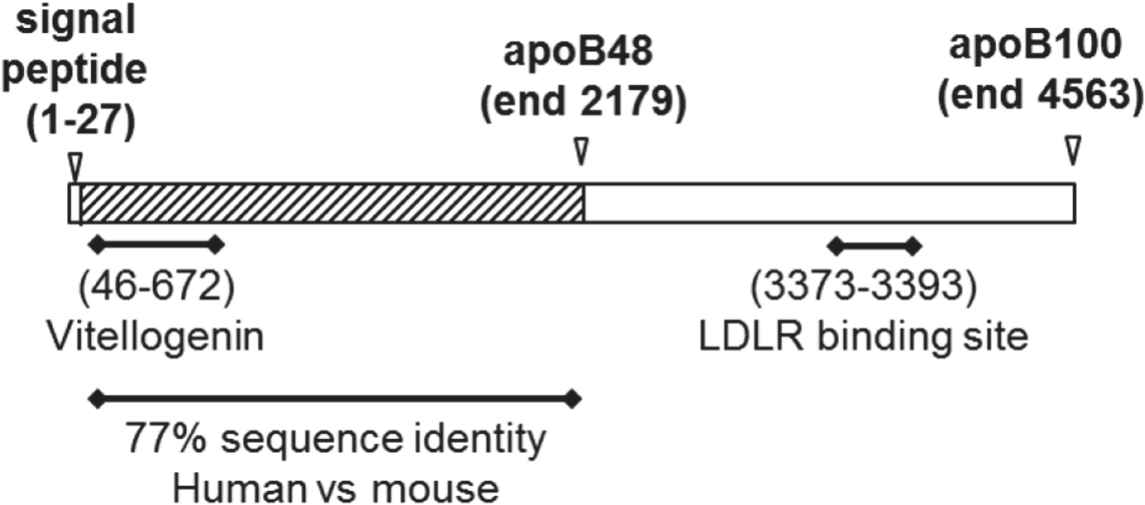
Depiction of apolipoprotein B. Schematic of human apoB100 (UniProt P04114) indicating the region aligning with vitellogenin, for which a crystal structure is available [57], the C-terminus of apoB48 and the low density lipoprotein receptor (LDLR) recognition site which facilitates uptake and clearance of apoB100 by the LDLR.

Lipoprotein particles are able to transport water insoluble lipids to peripheral tissues largely due to the inclusion of apolipoproteins. The formation of chylomicrons and the other forward cholesterol transport molecules, VLDL and LDL, requires the structural protein, apolipoprotein B (apoB). We have shown that apoB100, the 4536 amino acid protein essential for VLDL, and thus LDL, formation (Fig 1), limits pathogenesis caused by the Gram positive pathogen *Staphylococcus aureus* by disrupting virulence factor expression [10, 11]. *S*. *aureus* is both a commensal and an opportunistic pathogen which causes a broad range of diseases from skin and soft tissue infections (SSTI) to life threatening conditions in critically ill patients including pneumonia, endocarditis, osteomyelitis and bacteremia. The ability of *S*. *aureus* to cause disease in such varied host niches is facilitated by the use of two-component systems, including the accessory gene regulator (*agr*) quorum-sensing (QS) operon, to globally coordinate gene expression [12–22]. The *agr* operon coordinates a density-dependent switch from a colonizing to an invasive phenotype through the up-regulated expression of over 200 virulence factors such as proteases, lipases and toxins [23–25]. Activation of the *agr* system is mediated by binding of a secreted autoinducing peptide (AIP) to its cognate receptor, AgrC, on the bacterial surface. *S*. *aureus* isolates consist of four possible *agr* alleles, and AIP from each of the four alleles (AIP1-AIP4) differs in amino acid sequence and length, ranging from seven to nine amino acids. However, all AIPs share a common five-membered thiolactone ring which provides general structural similarity [12, 26]. We have shown that apoB100 binds to and sequesters *S*. *aureus* AIPs and therefore antagonizes *agr*-mediated virulence [10, 11]. In contrast to apoB100 in VLDL and LDL, chylomicrons contain a truncated form of apoB, apoB48, which is only produced in humans by intestinal enterocytes. The production of apoB48, the N-terminal 2152 amino acids (48%) of apoB100 (Fig 1), results from RNA editing by an enterocyte enzyme which introduces a premature stop codon in the coding region for apoB100 [27, 28]. Importantly, the association between enteral feeding and reduced risk of infectious complications compared to parenteral feeding in critically ill patients [8, 9] suggests a potentially unrecognized role for apoB48 in host innate defense. However, whether apoB48 is sufficient to antagonize *S*. *aureus agr*-signaling and pathogenesis has not been addressed.

To test the hypothesis that apoB48 is a host innate effector against *S*. *aureus* pathogenesis, we isolated apoB48-containing lipoprotein particles and assessed their ability to disrupt *agr*-activation. Here we report that apoB48 directly binds AIP and antagonizes *S*. *aureus* QS *in vitro*. IC50s for antagonism of *agr*-signaling are equivalent between apoB48 and apoB100, providing evidence that the AIP binding site is conserved between these apolipoproteins. Finally, exogenous apoB48 antagonized QS and inhibited morbidity *in vivo* in a mouse model of *S*. *aureus* infection. Therefore, this work provides proof that the AIP binding site is located in the N-terminus of apoB, demonstrates that apoB48 is sufficient to antagonize *S*. *aureus* QS, and suggests a previously unrecognized role for chylomicrons and intestinal enterocytes in human innate defense against bacterial pathogenesis.

## Results

### ApoB48 is sufficient to antagonize *S*. *aureus agr*-signaling *in vitro*

**Fig 2:**
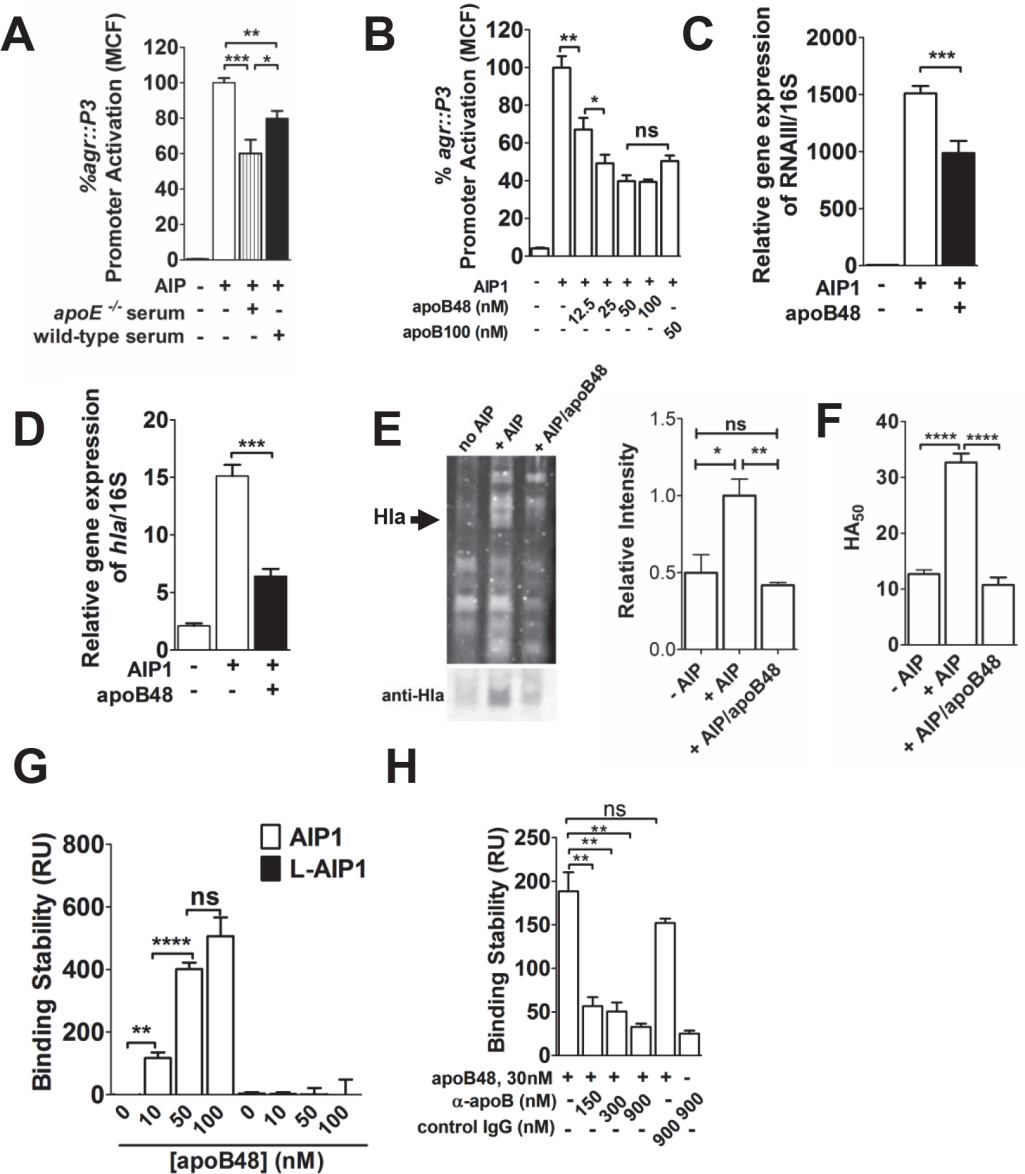
ApoB48-LP binds AIP and antagonizes *agr*-signaling. (A) *S*. *aureus agr*-I strain AH1677 (LAC) (2 x 10^7^ CFUs ml^-1^) was cultured for 2 hrs with 10 nM exogenous AIP1 and serum from *apoE* ^*-/-*^ or wild type (C57BL/6) mice (0.3%). *agr*::P3 promoter activation was measured by flow cytometry as mean channel fluorescence (MCF) and the MCF of the no serum control was normalized to 100%. (B) *S*. *aureus* was cultured as in (A) along with different concentrations of apoB48-LP or 50 nM human LDL (apoB100). *agr*::P3 promoter activation was measured by flow cytometry and the MCF of the no apoB control was normalized to 100%. Results shown are means ± SEM from three independent experiments performed in triplicate. (C-D) qRT-PCR analysis of (C) RNAIII and (D) *hla* expression relative to 16S rRNA under conditions described above. Results are means ± SEM from three independent experiments. (E) (Left) SDS-PAGE analysis of supernatants from 5 h cultures of LAC grown alone or with 50 nM AIP ± 50 nM apoB48-LP. Arrowhead indicates migration of Hla. (Right) Relative Hla concentration was determined by Western blot followed by quantification of band intensity compared to the + AIP control. Data are mean ± SEM of three experiments performed in triplicate. (F) Hla expression in supernatants grown as for (E), assessed via the rabbit red blood cell lysis assay. HA_50_ is the bacterial supernatant dilution factor required for lysis of 50% of the RBCs. Data are the mean ± SEM of triplicate experiments performed in duplicate. (G) Surface plasmon resonance (SPR) analysis of apoB48-LP binding to immobilized biotinylated AIP1. Binding was measured in resonance units (RU). (H) Anti-apoB antibody at 5-, 10- and 30-fold molar excess, but not IgG control, blocks apoB48-LP binding to AIP1. Results are the mean ± SEM of N = 3 to 5. ns, not significant; *, p<0.05; **, p<0.01; ***, p<0.001; ****, p≤0.0001.

We previously reported that apoB100, and not its associated lipids or other exchangeable apoproteins (apoA-I, apoC-I or apoE), binds and sequesters *S*. *aureus* AIP and antagonizes QS-dependent virulence [11]. To determine whether apoB48 was sufficient to inhibit *S*. *aureus* QS, we utilized serum from *ApoE* ^*-/-*^ mice as a ready source of apoB48-containing lipoprotein particles (apoB48-LP). Lipoproteins containing apoB100 are largely cleared by the LDL receptor (LDLR) which binds to the LDLR-binding sequence in the C-terminus of apoB100 (Fig 1). In contrast, clearance of apoB48 is mediated by apoE, an exchangeable apolipoprotein which facilitates uptake of chylomicron remnants via the LDLR and low-density lipoprotein related protein (LRP) [29–31]. Therefore, *ApoE* ^*-/-*^ mice amass circulatory apoB48-LP relative to wild-type mice. In order to determine whether apoB48-LP are capable of antagonizing QS, we first asked whether serum from *ApoE* ^*-/-*^ mice would antagonize *S*. *aureus agr* signaling *in vitro*. Because AIP binding to AgrC leads to transcriptional activation of the *agr* P3 promoter, we assessed *agr* signaling using an *agr* type I epidemic clone of community acquired-methicillin resistant *S*. *aureus* (CA-MRSA), USA300 LAC, with the *agr*::P3 promoter driving yfp expression [32]. As expected, at equivalent concentrations, serum (0.3%) from *ApoE* ^*-/-*^ mice showed increased inhibition of *agr*-signaling compared to serum from wild-type mice (Fig 2A). Since *ApoE* ^*-/-*^ serum is enriched in apoB48-LP compared to serum from wild-type controls, these results suggest that the increased apoB48-LP provided enhanced control of *S*. *aureus agr*-signaling in this system.

To confirm that apoB48-LP were responsible for the inhibition of *agr*-signaling by serum from *ApoE-/-* mice, we used size exclusion chromatography to purify apoB48-LP from the serum. ApoB48-LP eluted in the VLDL fraction which was highly enriched in serum from *ApoE* ^*-/-*^ mice compared to wild-type C57BL/6 mice (Panel A in S1 Fig). SDS/PAGE followed by Coomassie staining of the eluted fraction showed a single band at high molecular weight (227 kDa), consistent with the predicted molecular weight of apoB48 (~240 kDa), and Western blot analysis confirmed this band was apoB (Panel B in S1 Fig). No protein band was detected corresponding to the molecular weight of apoB100 (509 kDa). Additionally, dynamic light scattering analysis revealed a unimodal peak with a Z-average diameter of 72 nm, indicative of a homogeneous sample (Panel C in S1 Fig).

If apoB48-LP contributed to QS inhibition by serum from *ApoE* ^*-/-*^ mice, then we would expect strong inhibition of *agr*-signaling using purified apoB48-LP. As expected, apoB48-LP inhibited *agr*::P3 promoter activation in a dose-dependent manner (Fig 2B). Importantly, mouse apoB48 (UniProt E9Q414), which shares 77% sequence identity with the N-terminal 48% of human apoB100 (UniProt P04114), showed equivalent inhibition of *agr*-signaling compared to human LDL (apoB100-LP). Furthermore, apoB48-LP mediated QS inhibition was specific to *agr*-signaling, and not due to an effect on bacterial viability or suppression of bacterial growth (Panels D,E in S1 Fig). As would be predicted from the *agr*::P3 promoter activation assays, apoB48-LP antagonized AIP-induced expression of the P3-driven *agr* effector molecule, RNAIII, as well as *agr*-mediated expression of the gene encoding alpha-hemolysin, Hla, a key *S*. *aureus* virulence factor involved in invasive infection [33–39] (Fig 2C and 2D). Furthermore, apoB48-LP antagonized AIP-induced expression of Hla protein, as measured by both Western blot and rabbit RBC lysis functional assays (Fig 2E and 2F).

We previously demonstrated that the mechanism of apoB100-mediated inhibition of *agr*-signaling included direct binding to immobilized AIP [10, 11]. Based on our findings above, we predicted that apoB48-LP would likewise bind to AIP. To address this, we used surface plasmon resonance (SPR) to measure binding of apoB48-LP to immobilized AIP1. As expected, apoB48-LP bound immobilized, active (cyclized) AIP1 in a dose dependent manner, but did not bind the linear, biologically inactive form of the peptide (Fig 2G). In addition, the presence of antibody specific to apoB inhibited binding of immobilized AIP by apoB48-LP as previously shown for apoB100-LP [10] (Fig 2H). Furthermore, apoB48-LP bound immobilized AIP2, AIP3 and AIP4 in a dose dependent manner (Panels A-C in S2 Fig), and antagonized *agr*::P3 promoter activation by *agr-*II, *agr-*III and *agr-*IV clinical isolates (33) (Panels D-F in S2 Fig). These data demonstrate that a binding site for each of the four *S*. *aureus* AIPs is located within apoB48 and that apoB48-LP are sufficient to antagonize *agr*-signaling by isolates from each of the four *agr* alleles.

### ApoB48 and apoB100 are equally efficient inhibitors of *agr*-signaling

**Fig 3:**
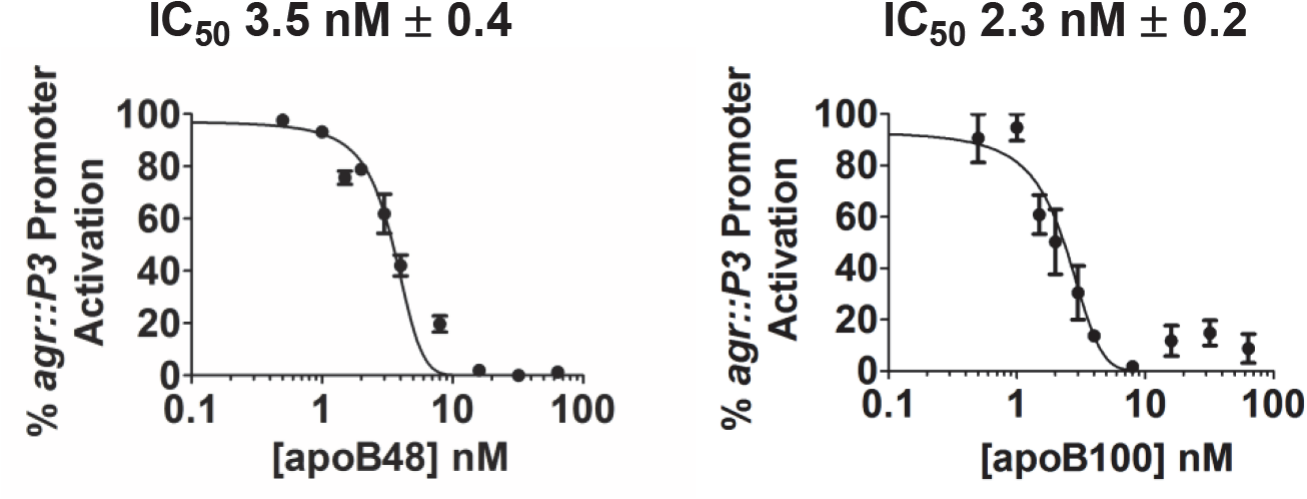
ApoB48- and apoB100-LP demonstrate equivalent inhibition of agr. AH1677 (2x10^7^ CFUs ml^-1^) was cultured with 50 nM AIP and increasing concentrations of mouse apoB48-LP or human LDL (apoB100). Data from three independent experiments are shown as normalized MCF versus the log of LP concentration.

The location of the AIP binding site within the N-terminus of apoB suggests that apoB48-LP would be an equally efficient antagonist of *agr*-signaling compared to apoB100-LP. To address this, we tested a range of equimolar concentrations of apoB48 and apoB100 for antagonism of *agr*-signaling driven by a fixed amount of exogenous AIP1. Inhibition dose-response curves for both apoB48-LP and LDL were saturable and therefore exhibited the characteristic response expected from a specific ligand-receptor interacting pair. Calculated IC50 values for apoB48- and apoB100-LP were essentially identical (3.5 and 2.3 nM, respectively (Fig 3)) and below the reported EC50 for AIP1-mediated *agr* activation (28 nM) [40]. These data demonstrate that a high-affinity binding site for AIP is located in the N-terminus of apoB and is conformationally conserved in apoB48.

### ApoB48 antagonizes *S*. *aureus* quorum sensing and pathogenesis *in vivo*

**Fig 4:**
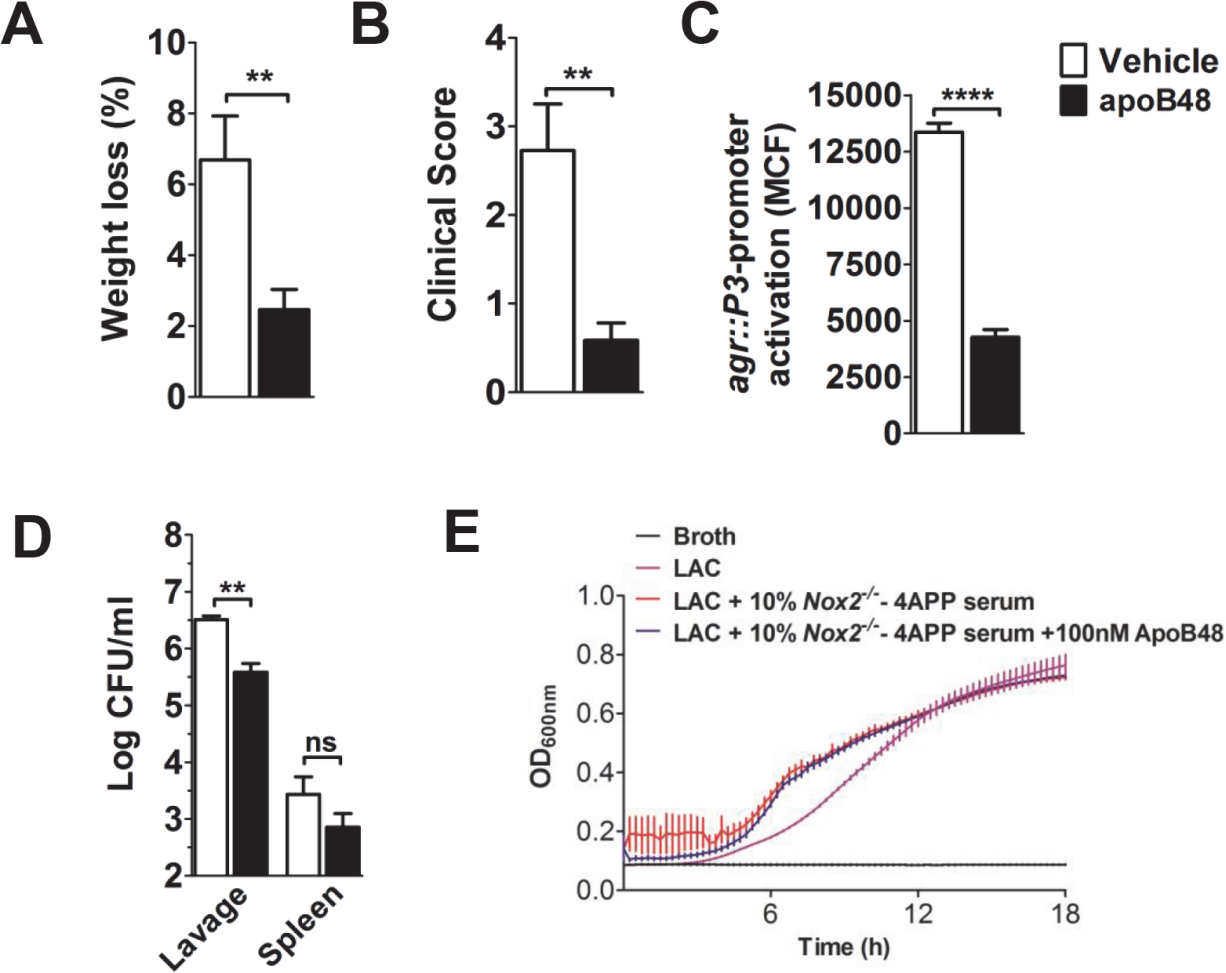
apoB48-LP antagonize *agr*-signaling and inhibit morbidity in an air-pouch model of *S*. *aureus* SSTI. AH1677 (3x10^7^ CFUs) and apoB48-LP (100 nM) or vehicle control, were injected into dorsal air-pouches of 4APP-treated, *Nox2* ^*-/-*^ mice. At 4 hrs post-infection, the mice were given a second dose of apoB48-LP or vehicle control. Twenty-four hrs post-infection, the following were determined: (A) percent weight loss; (B) clinical morbidity score (see Materials and Methods for details); (C) *agr*::*P3* promoter activation in pouch lavage; and (D) bacterial burden in the pouch lavage and spleen. Results are the mean ± SEM of N = 7 mice/group from two independent experiments (A,B) and N = 3 mice/group (C,D). (E) Growth curves of USA300 isolate LAC grown in broth or broth with 10% serum from 4APP-treated, *Nox2* ^*-/-*^ mice ± 100 nM apoB48-LP. Data shown are mean ± SEM from at least 2 independent experiments performed in duplicate. ns, not significant; **, p<0.01; ****, p≤0.0001.

Using a mouse air-pouch model of *S*. *aureus* skin and soft tissue infection (SSTI) (3, 4), we previously showed that apoB100-LP, LDL and oxLDL extravasate to the site of infection where they protect against *S*. *aureus agr*-dependent virulence [10]. Together with our *in vitro* data demonstrating that the AIP binding site is located within the N-terminal 48% of apoB100, we predicted that exogenous apoB48-LP would be sufficient to provide protection against *agr*-mediated pathogenesis *in vivo*. To demonstrate the potential protective role of apoB48 in critically ill patients, we utilized a hypolipidemic mouse model with high susceptibility to *S*. *aureus* infection. Specifically, we used mice lacking the phagocyte NADPH oxidase, Nox2, which were also treated with 4-aminopyrazole-(3,4-D)pyrimidine (4APP) to pharmacologically reduce serum lipoprotein levels [41, 42]. We chose *Nox2* ^*-/-*^ mice because chronic granulomatous disease patients lack Nox2 and are highly susceptible to *S*. *aureus* infection [43]. Using the air-pouch model of SSTI, we infected the mice by injecting USA300 LAC directly into the pouch along with either apoB48-LP or vehicle control. At twenty-four hours post-infection, apoB48-treated mice showed significantly reduced weight loss and a lower clinical score, both general measures of morbidity, compared to vehicle controls (Fig 4A and 4B). As expected, bacteria recovered in pouch lavage from apoB48-treated mice showed significantly reduced *agr*::P3 promoter activation compared to vehicle treated mice, demonstrating a significant reduction in *agr*-signaling (Fig 4C). Furthermore, apoB48-treated mice had decreased bacterial burden at the site of infection compared to controls (Fig 4D). Importantly, this was not due to a negative effect on *S*. *aureus* growth caused by the addition of apoB48-LP to serum from 4APP-treated *Nox2* ^*-/-*^ mice (Fig 4E). Having previously shown that reduction of serum lipoproteins negatively effects host clearance of *agr*+ but not *agr*- *S*. *aureus* [10, 11], and that pharmacological disruption of *agr*-signaling leads to enhanced immune cell-mediated bacterial clearance [44, 45], these data suggest that the reduced bacterial burden in apoB48 treated mice resulted from enhanced host mediated clearance following *agr*-inhibition. Together, these data demonstrate that apoB48 is sufficient to inhibit *agr*-signaling *in vivo* and to provide host protection against *S*. *aureus agr*-mediated pathogenesis.

## Discussion

Whereas serum lipoproteins are essential for lipid transport and homeostasis, pathologically elevated levels of LP are major contributors to cardiovascular disease and atherosclerosis [1, 46]. This deleterious role of LP is in stark contrast to their emerging role in host innate defense and the association between very low LP levels and increased susceptibility to the pathogenesis of infection [3–6, 47]. Here we extend our understanding of the contributions of LP to host innate defense by describing a role for apoB48 in limiting *S*. *aureus agr*-regulated pathogenesis. ApoB48 binds *S*. *aureus* AIP and antagonizes *agr*-signaling *in vitro* and *in vivo*, and because the *S*. *aureus agr* system coordinates expression of more than 200 virulence genes [25], this antagonism could provide broad protection against *S*. *aureus* infections facilitated by *agr*. Importantly, IC50 values for apoB48- and apoB100-mediated antagonism of *agr*-I-signaling were similar and lower than the reported EC50 for AIP1 activation of AgrC [40], suggesting that both forms of apoB can compete with AgrC for AIP binding. Because apoB48 in humans is solely produced by enterocytes during the uptake and packaging of dietary lipids into chylomicrons, our work may suggest a previously unrecognized role for chylomicrons and intestinal enterocytes in limiting the pathogenesis of *S*. *aureus* infections in critically ill patients.

**Fig 5:**
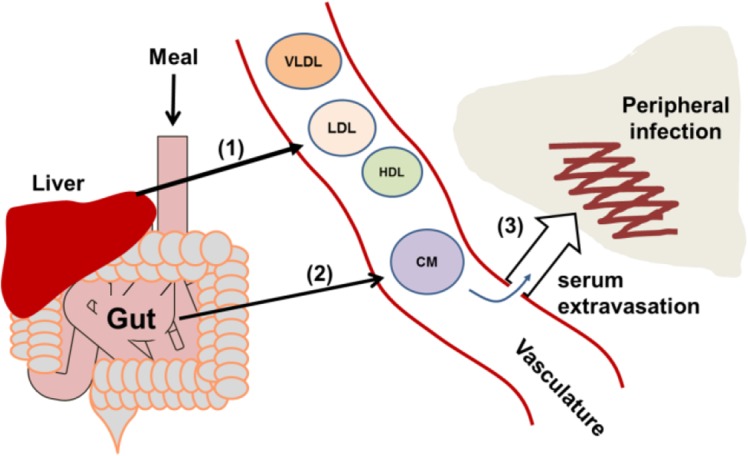
Model of lipoprotein access to sites of infection. (1) In a healthy subject, the liver releases HDL and VLDL, the latter of which is reduced to LDL by lipase activity. (2) Following oral feeding, enterocytes package dietary lipids and apoB48 into chylomicrons (CM). (3) Lipoproteins are available for host innate defense in the circulation or upon serum extravasation to sites of peripheral infection or inflammation. In a critically ill patient, LP release from the liver is limited as part of the APR and oral feeding may not be possible. The resulting reductions in serum LP levels may negatively impact both peripheral and systemic host innate defense against bacterial pathogens.

Although this is the first report describing a potential role for apoB48 and chylomicrons in innate defense against *S*. *aureus* QS, others have demonstrated roles for LP in host innate defense against this Gram positive pathogen, including LP binding to lipotechoic acid (LTA) as well as to certain staphylococcal toxins [48–50]. However, a broader role for LP in limiting bacterial pathogenesis lies in their ability to protect against potentially lethal endotoxic shock. LP, including chylomicrons, can bind lipopolysaccharide (LPS) from Gram negative pathogens, thus limiting LPS-induced production of pro-inflammatory cytokines and the resulting pathogenesis [51]. In this respect, we propose a model (Fig 5) whereby in a healthy subject, LP from the liver and chylomicrons from the gut are present in the vasculature from which they can act locally or extravasate to sites of peripheral infection or injury to contribute to host innate defense. In contrast, critically ill patients may have significant reductions in liver lipoprotein release during the APR. Therefore, although chylomicrons have a limited half-life in circulation [52], even a transient increase in serum lipoproteins could provide much needed defense against bacterial pathogenesis. If such patients are unable to receive nutrition perorally or via the gastrointestinal tract, needed for the formation of chylomicrons, they may have heightened susceptibility to bacterial pathogenesis. Moreover, all critically ill patients may have decreased intestinal motility resulting from anesthesia or opiate analgesia which could also impact a patient’s ability to form chylomicrons and thus their innate defense status [53, 54]. Although the full extent of the contribution of apoB and LP to host innate defense against bacterial pathogens is unknown, these studies suggest that the absence of dietary chylomicrons in critically ill patients could have previously unrecognized implications for their susceptibility to bacterial pathogenesis.

While here we describe a previously unrecognized role for apoB48, the importance of the N-terminus of apoB in host innate defense is not surprising considering the evolutionary structural and functional conservation with lipid transport molecules from other species, molecules which also contribute to innate defense [55]. A prime example of this is vitellogenin (Vg) which is produced in the liver of egg laying animals and cleaved to produce lipovitellin (LV) [56, 57]. Vg is a multivalent pattern recognition receptor (PRR) capable of binding LPS, peptidoglycan and LTA through distinct recognition sites [58], and thus is able to bind to *Escherichia coli* and *S*. *aureus* [56, 58, 59]. Vg/LV share greater than 30% sequence similarity with the N-terminus (17%) (Fig 1) of apoB and the crystal structure of LV is often used for structural modeling of apoB [57, 60–62]. Similarly, lipophorins, which are the insect version of lipoproteins, have two non-exchangeable proteins, apolipoprotein I and II (ApoLp-I, ApoLp-II) with homology to apoB [55, 63]. Recently, ApoLp from silkworm hemolymph was shown to bind to *S*. *aureus* cell surface LTA and inhibit the SaeRS two-component regulatory system [64, 65]. These findings in both vertebrate and non-vertebrate organisms suggest that LP and apoB-like proteins have an evolutionary role as dual purpose molecules in both lipid transport and host innate defense against bacterial pathogens.

It is important to note that this work does not provide evidence that a high-fat diet (HFD) and pathologically elevated chylomicron levels are beneficial. First, the detrimental effects of a high fat diet on overall health, including systemic inflammation, are well documented (reviewed in [66]). Second, animal studies have shown that mice chronically fed a HFD have decreased survival following intravenous *S*. *aureus* challenge compared to mice on a low-fat diet (LFD) [67]. Here, decreased survival was associated with an altered cytokine profile and decreased granulocyte phagocytic function relative to mice on the LFD. Rather, in critically ill patients with already reduced serum apoB100 levels, this work suggests that the lack of dietary chylomicrons could exacerbate their susceptibility to bacterial pathogenesis, particularly under conditions in which *agr*-signaling contributes to disease.

## Materials and Methods

### Reagents

Synthetic AIPs were purchased from Biopetide Co., Inc. (San Diego, CA). Human LDL was purchased from Biomedical Technologies, Inc., (Stoughton MA). Superose 6 10/300 prepacked columns were obtained from GE Healthcare Life Sciences. Other reagents were obtained as follows: rabbit anti-mouse apoB polyclonal antibody (LS-C20729, LifeSpan Biosciences); alkaline phosphatase conjugated anti-rabbit IgG (Sigma-Aldrich); nitro-blue tetrazolium and 5-bromo-4-chloro-3′-indolyphosphate (NBT/BCIP, Pierce), Modified Lowry Protein Assay Kit (Thermo Scientific); 3–8% Tris-Acetate protein gel (NuPAGE, Novex; Life Technologies); 4-aminopyrazole-(3,4-D)pyrimidine (4APP), (Sigma-Aldrich).

### Bacterial strains and culture

The *agr*::P3-yfp strains used in this study were generously provided by Dr. Alex Horswill (University of Iowa): AH1677 (*agr*-I isolate LAC), AH430 (*agr*-II isolate 502a), AH1747 (*agr*-III isolate MW2), and AH1872 (*agr*-IV isolate MN TG) [32]. Bacteria were grown in Trypticase Soy Broth (TSB) and early exponential phase bacteria were prepared as previously described [68]. CFU were determined by plating on TSA with 5% sheep blood (Becton, Dickinson and Company).

### ApoB48-lipoprotein particle purification

Serum from 12 week old male *ApoE* ^*-/-*^ mice on a C57BL/6 background was purchased from The Jackson Laboratory (strain B6.129P2-Apoetm1Unc/J). Serum was centrifuged to remove particulates and ≤ 600 μl aliquots were fractioned by gel filtration chromatography. Briefly, two Superose 6 10/300 prepacked columns were connected in series and equilibrated in PBS, pH 7.4 [69, 70]. One milliliter fractions were collected using an ÄKTA FPLC system with a flow rate of 0.3 ml min^-1^. The leading protein peak, identified by the absorbance at 280 nm, is the VLDL/apoB48-containing fraction. This fraction was pooled and concentrated.

Protein purity was assessed using SDS/PAGE (3–8% Tris-Acetate gel) and the protein identity was confirmed by Western blot using anti-mouse apoB polyclonal antibody and alkaline phosphatase conjugated anti-rabbit IgG. A FluorChem R system with AlphaView SA software was used for imaging (ProteinSimple, Santa Clara, CA). Protein concentrations of apoB48-LP and human LDL were determined using the modified Lowry Protein Assay. The hydrodynamic radius and particle size distribution of the purified fraction was determined by dynamic light scattering photon correlation spectroscopy (DLS) using a Malvern Zetasizer Nano-ZS at 25°C.

### *agr*::P3-yfp promoter activation assays

Early exponential phase, non-fluorescing bacteria (2x10^7^ ml^-1^) were incubated in TSB along with the indicated concentration of AIP, lipoprotein or vehicle control. Following 2 hours (unless otherwise noted) incubation with shaking at 37°C, bacteria were washed in PBS with 0.1% Triton X-100 by centrifugation at 1820 x g for 4 min at 4°C. Bacteria were then sonicated, cultured for CFU where indicated, and fixed for 5 minutes at room temperature in 10% buffered formalin phosphate. *agr*::P3 promoter activation was measured using the mean channel fluorescence (MCF) of induced YFP expression using an Accuri C6 (BD Accuri Cytometers, Ann Arbor, MI). Goat anti-human LDL and goat IgG in PBS were used in experiments for antibody reversal of apoB-mediated *agr* inhibition.

IC50 values were determined as follows. Fluorescence values were normalized by setting the highest mean value per replicate to 100%, and the lowest mean value per replicate to zero. IC50 values were calculated by fitting a normalized response–variable hill slope function to the data $y=100/(1+10^{({{(LogIC}_{50}-x)h)}_{\;}})$, where h is the Hill slope (GraphPad Prism 5.04). The positive feedback loop in the *agr* regulon necessitates the use of a variable Hill slope for these calculations.

### Growth curves

Growth curves were generated using the CLSI microbroth dilution method, with minor modifications. Briefly, LAC was diluted in Mueller Hinton II broth (MH; BD and Co., Sparks, MD) to 1 x 10^6^ CFU ml^-1^, confirmed by serial dilution plating on blood agar. Sera or lipoproteins were diluted in MH and mixed 1:1 in a 96-well plate to attain 5 x 10^5^ CFU ml^-1^ and the indicated concentration of serum or lipoprotein. Plates were incubated in an Infinite 200 plate reader (Tecan, Mannedorf, CH) at 37°C and maximum orbital shaking, for 18 h with OD_600_ measured every 15 min.

### qRT-PCR analysis

Cultures for quantitative RT-PCR were prepared as described above for *agr*::P3-yfp promoter activation assays. Bacterial RNA was isolated and purified using the Qiagen RNAprotect Bacteria Reagent and RNeasy Mini Kit (Qiagen, Valencia, CA). cDNA generation and qPCR analysis was carried out as previously described (3). RNAIII and *hla* were quantified relative to 16S RNA. Each experiment was performed in duplicate and samples assayed in triplicate using previously described primers and probes [10].

### Alpha-hemolysin (Hla) assays

LAC was cultured in 5 ml TSB for 5 h with DMSO, 50 nM AIP, or 50 nM AIP with 50 nM apoB48-LP. Bacteria were pelleted by centrifugation and supernatants were filtered through a 0.2 μm HT Tuffryn membrane (Pall, Port Washington, NY). Supernatants were diluted to equivalent protein concentrations based on A_280_ (Nanodrop 1000; Thermo Fisher Scientific Inc., Grand Island, NY). Briefly, supernatant (~9 μg protein) was separated by SDS-PAGE on a Bolt 4–12% BisTris gel (Thermo Fisher Scientific Inc.) and either stained with SYPRO Ruby (Lonza, Rockland, ME) according to manufacturer’s directions, or transferred to a polyvinylidene fluoride membrane. Membranes were blocked for 2 h at 22°C with TBST (20 mM Tris [pH 7.5], 150 mM NaCl, 0.1% Tween 20) and 5% nonfat dry milk. Membranes were probed with anti-Hla antibody (ab15948; Abcam, Cambridge, MA) at a 1:1,000 dilution, washed with TBST, and Hla detected with alkaline phosphatase secondary antibody (ab97127; Abcam). Bands were developed with nitroblue tetrazolium / bromo-chloro-indolyl-phosphate (NBT/BCIP) for 15 min at 22°C. Band relative intensities compared to the + AIP control were measured using ImageJ software [71].

The rabbit red blood cell lysis assay was performed as previously described [72], with minor modifications. Serial two-fold dilutions of filter supernatants were incubated at 37°C for 1 h in a 4% solution of rabbit red blood cells (rRBCs). Lysis was assessed spectrophotometrically at OD_650_. Data were analyzed by non-linear regression fit to a four-parameter logistic curve and represented as the HA_50_. The HA_50_ is 1/dilution number required for 50% rRBC lysis compared to a 0.1% Triton X-100 control.

### AIP binding analysis

A Biacore X100 surface plasmon resonance instrument (Biacore Life Sciences, GE Healthcare) was used to assess lipoprotein binding to N-terminally biotinylated AIPs immobilized on a streptavidin chip as previously described [10, 11]. Binding stability is reported as the resonance units (RU) from the binding of the lipoprotein to the immobilized AIP ligand minus the RU from the lipoprotein binding to the unmodified reference surface. Polyclonal rabbit anti–mouse VLDL antibody and rabbit IgG in PBS were used in experiments for antibody reversal of lipoprotein binding to AIP.

### Mouse infection model

Animal studies were conducted in strict accordance with recommendations in the Guide for the Care and Use of Laboratory Animals, the Animal Welfare Act and U.S. federal law. The protocol was approved by the University of New Mexico Health Sciences Center Institutional Animal Care and Use Committee (IACUC) (approval number 14-101174-HSC). All infections were performed under anesthesia (inhaled isoflurane) and all efforts were made to minimize suffering. Mice were euthanized by carbon dioxide inhalation. Male, 8–12 week old *Nox2* ^*-/-*^ mice on the C57BL/6 background [43] were purchased from Jackson Laboratory (Bar Harbor, ME). Subcutaneous air pouches were created on the backs of the mice as previously described [10, 11, 68, 73–77]. At 48, 24 and 0 hours before infection, the mice were injected i.p. with 4-aminopyrazole-(3,4-D)pyrimidine (4APP) (500 μg) or buffer control. Subcutaneous air pouches were infected with 3x10^7^ CFU of AH1677. Twenty-four hours post infection, weight loss was measured and morbidity scored according to the following clinical scale: appearance: 0–4; natural behavior: 0–3; hydration status (skin pinch test): 0–3; provoked behavior: 0–4. The overall clinical score is the sum of the scores in the 4 categories with a maximum of 14. Pouch lavage and spleen CFU were determined by plating on blood agar. In addition, *agr*::P3 promoter activation of bacteria from the pouch lavage was measured as MCF as described above.

### Statistical analyses

Data are shown as mean ± SEM. Statistical analysis of all *in vitro* data was performed with the two-tailed Student’s t-test. *In vivo* data were analyzed by the Mann-Whitney U test for non-parametric data.

## Supporting Information

S1 Fig Purification of apoB48-LP from serum of *ApoE* ^*-/-*^ mice. (A) Fractionation of serum from *ApoE* ^*-/-*^ or wild-type mice by size exclusion chromatography. Major lipoprotein containing peaks are indicated. ApoB48-containing LP were eluted in the VLDL fraction. (B) SDS-PAGE and Western blot of *ApoE* ^*-/-*^ serum and the pooled VLDL fraction containing highly purified apoB48-LP. (C) Dynamic light scattering (DLS) analysis of purified apoB48-LP showing a unimodal size distribution peak. (D) Bacterial count of AH1677 grown in the presence or absence of 50 nM apoB48-LP from experiment shown in Fig 2D. (E) Growth curves of USA300 isolate LAC grown in broth, or broth with either 100 nM or 0.1 nM purified apoB48-LP added. Data shown are mean ± SEM from at least 2 independent experiments performed in duplicate.
(TIF)

S2 Fig apoB48-LP bind AIP2-AIP4 and antagonize *agr*-signaling all *agr* alleles. SPR analysis of apoB48-LP binding to immobilized (A) AIP2, (B) AIP3 and (C) AIP4. (D-F) *agr*::P3 promoter activation assay with the indicated strain at 2x10^7^ CFUs ml^-1^, 50 nM of the appropriate exogenous AIP and 50 nM apoB48-LP: (D) AH430 (*agr*-II, 5 hrs); (E) AH1747 (*agr*-III, 4.5 hrs) and (F) AH1872 (*agr*-IV, 2 hrs). Results are the mean ± SEM from triplicate experiments. ns, not significant; *, p<0.05; **, p<0.01; ***, p<0.001; ****, p≤0.0001.
(TIF)
