# Supplementary figures and images for: Apolipoprotein B48, the Structural Component of Chylomicrons, Is Sufficient to Antagonize Staphylococcus aureus Quorum-Sensing
Source: PLoS One. 2015 May 5;10(5):e0125027. doi: 10.1371/journal.pone.0125027 (PMC4420250; doi:10.1371/journal.pone.0125027)

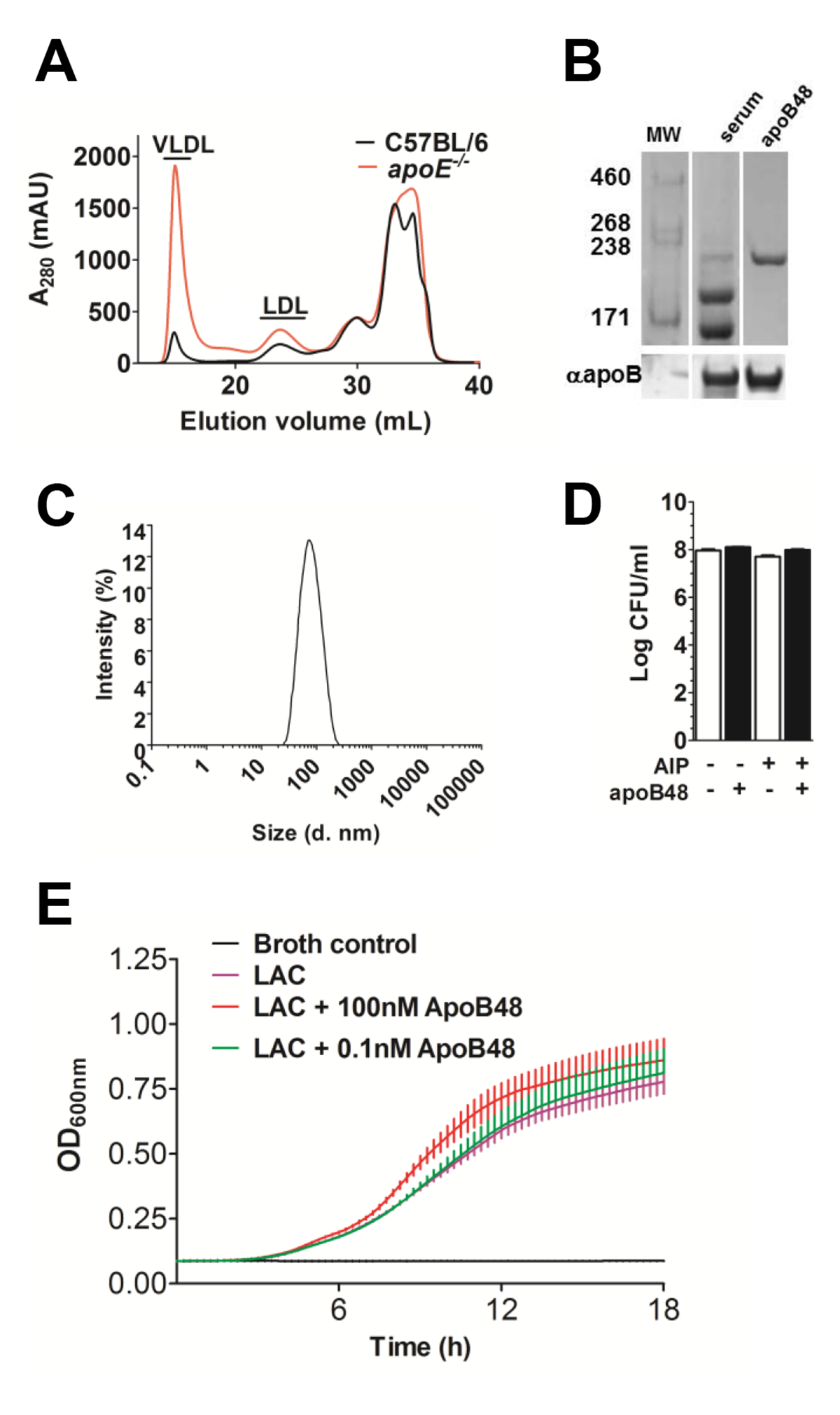

Supplement: S1 Fig — (A) Fractionation of serum from ApoE -/- or wild-type mice by size exclusion chromatography. Major lipoprotein containing peaks are indicated. ApoB48-containing LP were eluted in the VLDL fraction. (B) SDS-PAGE and Western blot of ApoE -/- serum and the pooled VLDL fraction containing highly purified apoB48-LP. (C) Dynamic light scattering (DLS) analysis of purified apoB48-LP showing a unimodal size distribution peak. (D) Bacterial count of AH1677 grown in the presence or absence of 50 nM apoB48-LP from experiment shown in Fig 2D. (E) Growth curves of USA300 isolate LAC grown in broth, or broth with either 100 nM or 0.1 nM purified apoB48-LP added. Data shown are mean ± SEM from at least 2 independent experiments performed in duplicate. (TIF) [file pone.0125027.s001.tif]

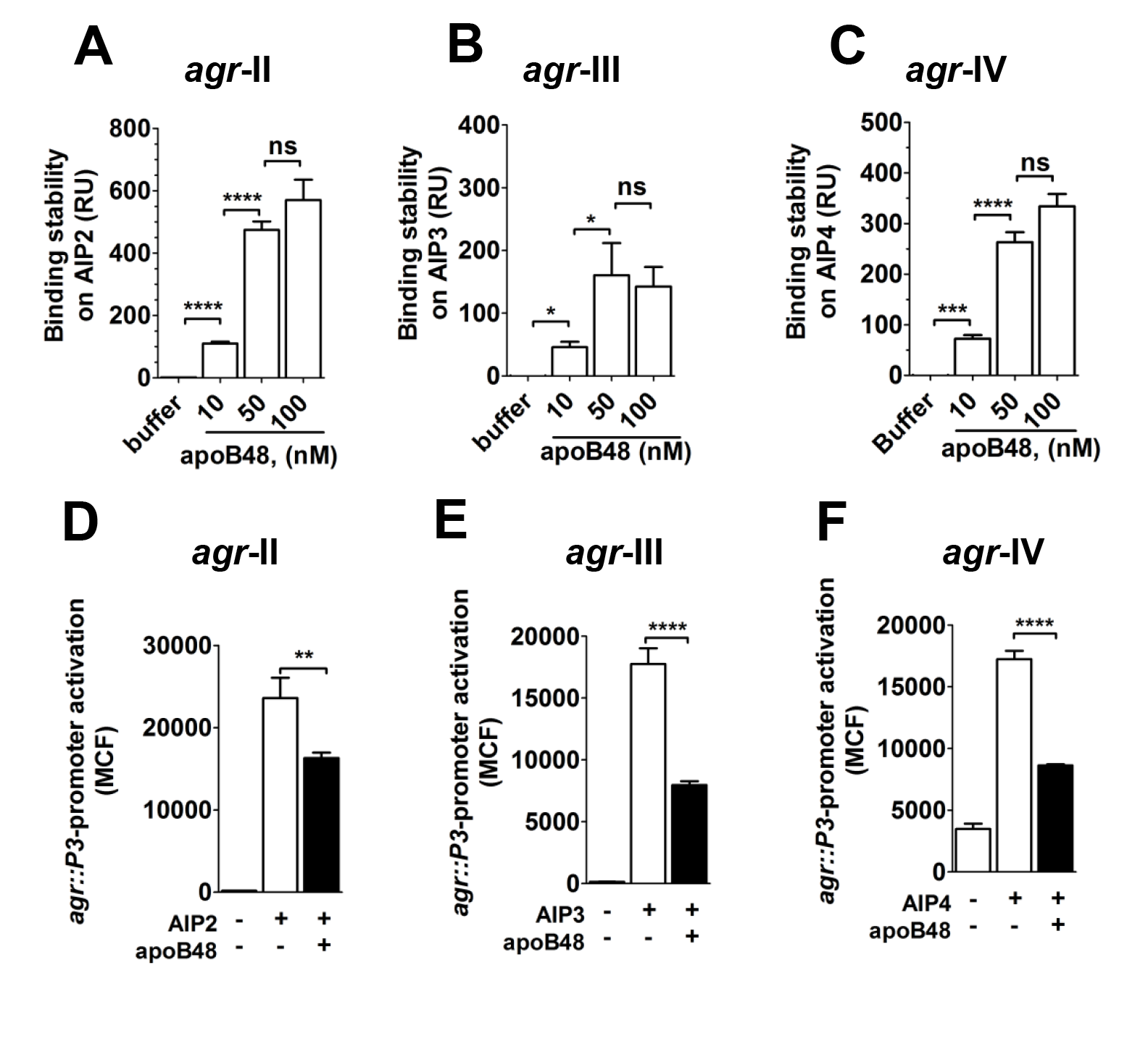

Supplement: S2 Fig — SPR analysis of apoB48-LP binding to immobilized (A) AIP2, (B) AIP3 and (C) AIP4. (D-F) agr::P3 promoter activation assay with the indicated strain at 2x10^7^ CFUs ml-1, 50 nM of the appropriate exogenous AIP and 50 nM apoB48-LP: (D) AH430 (agr-II, 5 hrs); (E) AH1747 (agr-III, 4.5 hrs) and (F) AH1872 (agr-IV, 2 hrs). Results are the mean ± SEM from triplicate experiments. ns, not significant; *, p<0.05; **, p<0.01; ***, p<0.001; ****, p≤0.0001. (TIF) [file pone.0125027.s002.tif]
